# Plasmid-based gap-repair recombineered transgenes reveal a central role for introns in mutually exclusive alternative splicing in *Down Syndrome Cell Adhesion Molecule* exon 4

**DOI:** 10.1093/nar/gky1254

**Published:** 2018-12-12

**Authors:** Irmgard U Haussmann, Pinar Ustaoglu, Ulrike Brauer, Yash Hemani, Thomas C Dix, Matthias Soller

**Affiliations:** 1 School of Biosciences, College of Life and Environmental Sciences, University of Birmingham, Edgbaston, Birmingham B15 2TT, UK; 2 School of Life Science, CSELS, Coventry University, Coventry CV1 5FB, UK

## Abstract

Alternative splicing is a key feature of human genes, yet studying its regulation is often complicated by large introns. The *Down Syndrome Cell Adhesion Molecule* (*Dscam*) gene from *Drosophila* is one of the most complex genes generating vast molecular diversity by mutually exclusive alternative splicing. To resolve how alternative splicing in *Dscam* is regulated, we first developed plasmid-based *UAS* reporter genes for the *Dscam* variable exon 4 cluster and show that its alternative splicing is recapitulated by *GAL4*-mediated expression in neurons. We then developed gap-repair recombineering to very efficiently manipulate these large reporter plasmids in *Escherichia coli* using restriction enzymes or sgRNA/Cas9 DNA scission to capitalize on the many benefits of plasmids in phiC31 integrase-mediated transgenesis. Using these novel tools, we show that inclusion of *Dscam* exon 4 variables differs little in development and individual flies, and is robustly determined by sequences harbored in variable exons. We further show that introns drive selection of both proximal and distal variable exons. Since exon 4 cluster introns lack conserved sequences that could mediate robust long-range base-pairing to bring exons into proximity for splicing, our data argue for a central role of introns in mutually exclusive alternative splicing of *Dscam* exon 4 cluster.

## INTRODUCTION

Alternative splicing (AS) is a major mechanism to generate vast proteomic diversity from the limited number of genes present in higher eukaryotes (1,2). In humans, ∼95% of genes harbor AS, while 63% of *Drosophila* genes have AS (3). Alternative splicing is a highly regulated process and its miss-regulation is a major cause of human disease (4–7).

**Figure 1.**
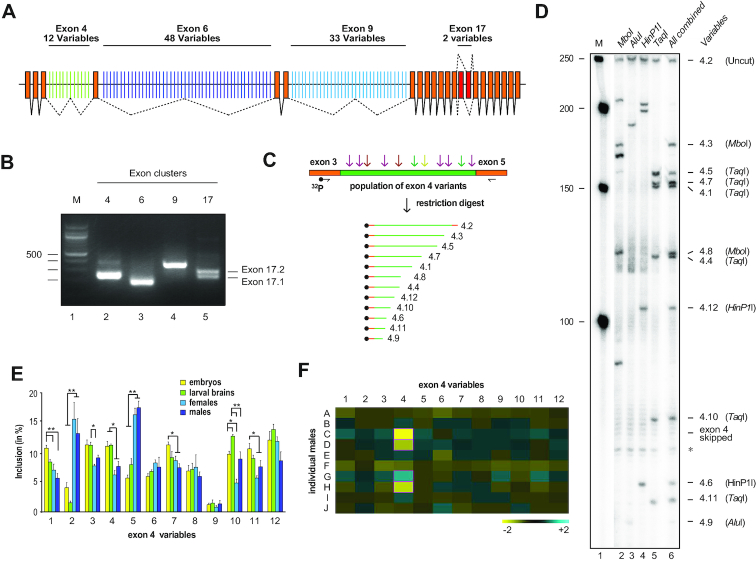
Analysis of *Dscam* exon 4 mutually exclusive splicing during development and in individual flies. (**A**) Schematic of the *Dscam* gene depicting constitutively spliced (orange) and variable exons in clusters 4 (green), 6 (dark blue), 9 (light blue) and 17 (red), where by mutually exclusive alternative splicing only one variable exon is chosen. (**B**) RT-PCR products for the variable exon clusters shown on an agarose gel. Note that only exons for the variable exons 17 can be resolved. One hundred base pair size markers are shown on the left. (**C**) Schematic of the method used to resolve inclusion levels of variable exons using a ^32^P labeled primer and a set of restriction enzymes followed by separation on a denaturing acrylamide gel. (**D**) Denaturing acrylamide gel showing inclusion of individual exon 4 variable exons after identification by restriction digest of individual enzymes (MboI, AluI, HinPI and TaqI, lanes 2–5) and the combination thereof (lane 6). Size markers (M) are shown on the left. (**E**) Developmental profile of inclusion levels of exon 4 variables in embryos (yellow), third instar larval brains (green), adult females (dark blue) and males (light blue) shown as means with standard error from three experiments. Statistically significant differences are indicated above bars (*P < 0.05, **P < 0.01). (**F**) Mean inclusion levels of exon 4 variables from ten individual adult males (A–J) are shown as fold change from the total mean. Blue indicates increased, and yellow reduced inclusion, respectively. Statistically significant differences are indicated by purple borders (*P* < 0.05).

One of the most complex genes in regard to AS is the *Down Syndrom Cell Adhesion Molecule* (*Dscam*) gene from arthropods. In *Drosophila Dscam*, 36 012 isoforms can be made by mutually exclusive AS in four variable clusters consisting of 12 (exon 4), 48 (exon 6), 33 (exon 9) and 2 (exon 17) variables (Figure 1A). *Dscam* in *Drosophila* directs neuronal wiring and phagocytosis in the immune response, but little is known how AS in this gene is regulated (8–10).

AS is regulated by RNA binding proteins (RBPs), which recognize regulatory sequences in exons and intervening non-coding introns. These regulatory sequences consist of short binding motifs, which are, however, highly degenerate at a genomic scale (3,11,12). Currently, we have only a very limited understanding about the sequence codes used by RBPs and other factors for identifying splice sites (ss) and regulate AS with high fidelity in a complex cellular environment (12–16). Elucidating this splicing code to make accurate predictions about the outcome of AS in different cell types and conditions requires reporter genes, in which all regulatory sequences can be incorporated and efficiently manipulated.

Traditionally, plasmid-based reporters have been used for the analysis of AS, but with an increasing size of plasmids, their manipulation by standard cloning procedures becomes extremely difficult or even impossible, which is further aggravated by the mostly large sizes of introns like in *Drosophila Dscam*. To capitalize on the many benefits of plasmids for cloning and transgenesis, we developed highly efficient gap-repair recombineering for plasmids in *E. coli*.

Homologous recombination in *E. coli* (recombineering) provides a versatile alternative to manipulate DNA, particularly in large Bacterial Artificial Chromosomes (BACs) and viruses (17–21), but has received little attention for the manipulation of plasmids (22). Recombineering applications rely on phage proteins, e.g. the *Red* operon from λ phage containing Red α, Red β and Red γ, which are used either integrated into the *E. coli* genome or provided as a plasmid (20,23). Red α is a 5′-3′ exonuclease leading to single stranded ends, Red β is a single stranded annealing protein and Red γ inhibits RecBCD exonuclease. Homologous recombination is initiated by a double strand break, which is then resected by Red α leading to annealing of the two single stranded regions. Transfection of a linearized plasmid containing homology regions on either side into a BAC containing *E. coli* in combination with the expression of the λ Red proteins has become a standard method to sub-clone sequences from large BAC clones into plasmids (18).

Concentrations of transcripts and trans-acting factors are critical parameters in the regulation of AS (13). To allow for robust comparison of AS splicing reporters harboring mutations in regulatory elements, they need to be inserted into the genome at the same genomic location to normalize for position effects. In *Drosophila*, phiC31 integrase has been employed for site-specific integration into the genome (24–26), but phiC31 mediated integration or similar systems are now also becoming widely used in various other model organisms and cell lines (27–30). In phiC31 mediated integration, insertion of reporter constructs is mediated by recombination between the short *attP* sequence in the plasmid and an *attB* sequence in the genome, previously inserted by transposon-mediated transformation. A further advantage of *Drosophila* to study tissue-specific AS is the binary *GAL4/UAS* system and the availability of numerous *promoterGAL4* lines (31–33), which when crossed to *UAS* containing reporter transgenes will result in tissue-specific or cell-type specific expression.

Here, we have developed a simple and efficient plasmid-based AS analysis pipeline using *Drosophila Dscam* as a model gene for transgene analysis, yet applicable to other model organisms and cell lines using phiC31 mediated integration. To resolve alternatively spliced exons with the same size, we make use of their sequence differences to distinguish them by digestion with restriction enzymes providing a cost-effective analysis tool. Accordingly, all 12 variable exons from the *Dscam* exon 4 cluster can be separated on denaturing acrylamide gels. To be able to analyse *Dscam* AS regulation tissue-specifically in neurons, we designed a plasmid-based transgenic reporter system using heterologous expression by the *GAL4/UAS* system. To efficiently introduce modifications in these large reporter plasmids, we employed λ Red protein mediated homologous recombination in *E. coli*. In contrast to classic cloning, *E. coli* mediated recombineering is robust over a broad concentration range of fragments and highly efficient (up to 100%) for large plasmids up to 22 kb in addition to having a very low error rate. Furthermore, limitations in finding rare-cutting restriction enzymes can be overcome by in vitro sgRNA/Cas9 mediated DNA scission. Our results from the analysis of *Dscam* exon 4 alternative splicing indicate that inclusion frequency of variables differs little during development and between individual flies, and is to a large degree determined by sequences harbored in variable exons. In contrast, introns are required for inclusion of both proximal and distal exons. Since robustly conserved sequences in introns of the variable exon 4 cluster are absent our data argue against a long-range base-pairing mechanism that brings ss into proximity for exon 4 selection. Hence, our data show a key role for introns, possibly through cluster-specific RNA binding proteins (59), in selection of variable exons in mutually exclusive alternative splicing in the *Dscam* exon 4 cluster.

## MATERIALS AND METHODS

### RNA extraction, RT-PCR, restriction digestion, denaturing acrylamide gels, western blots and splice site analysis

Total RNA was extracted using Tri-reagent (SIGMA) and reverse transcription was done with Superscript II (Invitrogen) according to the manufacturer's instructions using either primer Dscam 11RT1 (CGGAGCCTATTCCATTGATAGCCTCGCACAG, 1 pmol/20 μl reaction) for endogenous *Dscam* or ewgpART1 (GCCGTAATTGACTAGATTC, 10 fmol/20 μl reaction) for transgenes. PCR was done with primers 3F3 (GCAACCAGTTCGGAACCATTATCTCCCGGGAC) and 5R1 (CCAGAGGGCAATACCAGGTACTTTC) for 37 cycles with 1 μl of cDNA to amplify endogenous Dscam transcripts. To amplify *Dscam* from transgenes by nested PCR, we used primers Dscam ex4end F1 (GCATCGCTAGCTAGTCAGACCCTAGCTGCCAATCCCCCAG) and Dscam RT9 (GGCCTACTAGTCGTCGGCTGGTCGC) for 25 cycles with 1 μl of cDNA in a 50 μl reaction, and then for 22 cycles with primers 3F1 and 5R1 using 5μl of the first PCR in a 50 μl reaction. Primers were labeled with ^32^P gamma-ATP (6000 Ci/mmol, 25 μM, Perkin Elmer) with PNK (NEB) to saturation and diluted as appropriate. From a standard PCR reaction with a ^32^P labeled primer, 10–20% were sequentially digested with a mix of restriction enzymes (NEB) according to their buffer requirements, afterwards phenol/chloroform extracted and precipitated, and analyzed on standard 6% sequencing type denaturing polyacrylamide gels. After exposure to a phosphoimager (BioRad), individual bands were quantified using ImageQuant (BioRad) and inclusion levels for individual variable exons were calculated from the summed up total of all variables. Statistical analysis was done by a two tailed-t-test or for multiple comparisons by one-way ANOVA followed by Tukey–Kramer *post-hoc* analysis using graphpad prism. Inclusion levels of exon 4 variables of individual males were transformed into fold-change and fitted into a Gaussian distribution to determine variable exons with significantly different inclusion levels. Heat-maps were generated by uploading tab delimited text files to Matrix2png interface (34). RT from *D. virilis* total RNA was done with primer vDscam 11RT1GSP (GCCGATGCCGTTGATGGCCTCACACAAGTAG) and PCR of Dscam exon 4 with primers vDscam 3F1 (GCAACCAGTTTGGCTCAATCATATCC) and vDscam 5R2 (GCCGGAGGGCAGAACGAGGTATTTG). PCR products were digested with TaqI and HaeIII. Exon specific primers were selected from the most diverge regions of variable exons based on an alignment (Clustal W and/or Clustal V) and were as follows. Dscam 3F4 (CCAGGAGGTCCATGCCCAGGTGTAC) was used in combination with Dscam 4.5R1 (CCGGAGCGTACTCAGTGCCGTCACTG), Dscam 4.6R1 (GTTCTCAGAGGGACGCAGTTCGGTG) or Dscam 4.7R1 (CGTAATTGTCCGAAAAGGACAAGACATTG), and Dscam 5R2 (CTGTGATGACCAATCGTCCTTTTGTGGCAC) was used in combination with Dscam 4.5F1 (GACGGCACTGAGTACGCTCCGGAAGAG), Dscam 4.6F1 (GCACCGAACTGCGTCCCTCTGAGAAC) or Dscam 4.7F1 (CAATGTCTTGTCCTTTTCGGACAATTACG). Western blots were done as described using anti-HA antibodies (1:50, MAb 3F10, Roche) (35). Analysis of ss strength was done with MaxEnt Splice Site Scoring server provided by the Burge lab at MIT (http://genes.mit.edu/burgelab/software.html) (36). Vista alignments were generated as described (14).

### Retrieval of genomic sequences from BAC clones

Homology arms encompassing the ends of the *Dscam 3–5* constructs were PCR amplified with primers Dscam ex4end F1 and R1 (GCATCGCTAGCtAGTCAGACCCTAGCTGCCAATCCCCCAG and ATCAGGGCAGTGCAAAGTAGTCACCTGTTG), and Dscam ex4end F2 and R2 (ATCCTTAATCATTTCAAAGTCACATTGCATGGTCAACG and CCTACTAGTCGTCGGCTGGTCGCGGCCGCCCGTACGTCCTTTTGTGGCACTTAATCGGG), and cloned into Nhe I and Not I cut *pUC 3GLA UAS HAi* (generated by standard cloning methods, accession number KM253740), in a three way ligation generating an EcoRV site such that the split sites exactly matches the genome sequence at the end/beginning of the homology arms. Retrieval of genomic *Dscam* from the BAC clone (CH321–83C24, BACPAC resources, carrying a chloramphenicol resistance) was done by electroporating the *pSC101-BAD-gbaA* plasmid (encoding the λ Red and *E. coli* RecA proteins and carrying a tetracycline resistance, Genebridges) (23) into the BAC containing *E. coli*, expressing the recombineering proteins and transfection with the linearized retrieval vector.

Electro-competent BAC harboring cells were generated by inoculating 1 ml of LB with 10 μl of an overnight culture and grown to an OD_600_ of 0.6 (∼6 h). Cells were then pelleted with 10 000 g for 30 seconds at 0°C and washed with 10% glycerol (UltraPure, Invitrogen). After repeating this step, cells were electroporated with 1 ng of *pSC101-BAD-gbaA* plasmid (1 mm cuvettes using 1.8–2.5 V at 25 μFD capacitance, 200 Ω controller and 125 μFD extender, BioRad Gene Pulser) and grown overnight at 30°C.

Recombineering-competent BAC harboring cells were generated by inoculating 1 ml of LB with 10 μl of an overnight culture and grown to an OD600 of 0.2 (∼2 h) at 30°C. 10 μl 10% l-arabinose was added and the cultures put into a 37° shaking incubator to induce expression of the recombineering proteins until OD600 of 0.35–0.4 (about 1 h). At 37°C, DNA replication and partitioning to daughter cells of the *pSC101-BAD-gbaA* plasmid is inhibited (37). The cells were then pelleted with 10 000 g for 30 s at 0°C and washed with 10% glycerol for two times, and electroporated with 10 ng of linarized plasmid and plated on ampicilin plates. Retrieval efficiency with 300–600 bp homology arms generally is very high (60–90%), but we have also obtained sequences from BAC clones with 60–80 bp homology arms, although with a much lower efficiency (5–10%).

### Generation of recombination protein expressing competent *E. coli* for gap-repair recombineering

Chemical competent cells (DH5alpha or EPI 300, Epicentre) for gap-repair recombineering were generated according to Hanahan *et al.* (38) by transfection with the *pSC101-BAD-gbaA* plasmid, and then grown and induced with l-arabinose in a larger volume as described above. Cells were cooled on ice for 15 min, then pelleted at 4°C for 15 min at 3000 g, resuspended in 33 ml (for 1 l culture) of ice cold buffer RF1 (100 mM RbCl, 50 mM MnCl_2_, 10 mM CaCl_2_, 80 mM potassium acetate pH 5.8, 15% UltraPure glycerol) and incubated for 1 h on ice. After pelleting, the cells were resuspended in 2 ml ice cold buffer RF2 (10 mM MOPS pH 6.8, 10 mM RbCl, 75 mM CaCl_2_, 15% UltraPure glycerol), incubated for 15 min on ice and aliquots put to −80°C for storage. Transformation efficiency was determined with a 3 kb plasmid and was between 10^5^ to 10^6^ transformants/μg DNA. Electro-competent cells (EPI 300, Epicentre) for recombineering of larger BAC clones were prepared by transfection of cells with the *pSC101-BAD-gbaA* plasmid, and grown in 500 ml LB inoculated with a 10 ml overnight starter culture to 0.2 OD_600_ at 21°C as adapted from Novakova *et al.* (39). After induction with L-arabinose (0.3% w/v final) for 2 h (until 0.35–0.4 OD_600_), cells were pelleted at 4°C for 15 min at 800 g and washed three times with 10% UltraPure glycerol (v/v, Invitrogen), resuspended in 1 ml 10% UltraPure glycerol and frozen at −80°C. After electroporation, cells were inoculated in LB media supplemented with 5 mM MgCl_2_ for 45 min at 37°C before plating.

### Gap-repair recombineering and *Drosophila* phiC31 integrase-mediated transgenesis

Inserts 4.6/9.8 and 4.8/9.8 were cloned by fusing two overlapping PCR products with flanking primers and combining with the second fragment and the *pOT2* vector (BDGP, chloramphenicol resistant) in a three way ligation or by Gibson assembly (NEB)(40). To cut the insert out from the *pOT2* vector SmaI sites were incorporated on either site such that the last nucleotides of the insert exactly match the sequence from *Dscam*. The ampicilin-resistance destination plasmid *pUC 3GLA UAS HAi Dscam 3–5* was cut with Sfo I and PshA I, and the insert containing chloramphenicol-resistant *pOT2* plasmid with Sma I, extracted with phenol/chloroform and precipitated. Vector and insert were then mixed without prior gel-purification (best results were generally obtained with 50 ng of vector and a molar excess of insert over vector). Gel-purification of the fragments is not required, because the two plasmids have a different antibiotic selection and efficient recombination can only occur between the desired fragments. The vector/insert mix was then incubated for 30 min at 50°C and added to 25 μl recombineering competent cells. After a 15 min incubation on ice, cells were heat-shocked (2 min in a 42°C water bath), put on ice for another minute, SOC media added and plated on ampicillin agar plates after a 30–45 min incubation at 36°C. Large plasmids were propagated at 36°C as temperature >37° resulted in undigestable DNA and aberrant plasmids.

For phiC31 mediated transformation, constructs were injected into y^1^ w* M{vas-int.Dm}ZH-2A; PBac{y+-attP-3B}VK00013 with the landing site inserted at 76A as previously described (41). Prior to insertion of GFP marked constructs, the GFP and RFP markers had been removed in y1 w* M{vas-int.Dm}ZH-2A by Cre mediated recombination (25). Efficient removal of the *3xP3 GFP* marker by Cre has been validated in transgenes. We noted the *3xP3 GFP* marker is expressed at variable strength in the eye depending on landing site and construct and for some constructs screening for transformants on the day of eclosion was essential to see the weak GFP marker in the eye. Although the marker can be weakly expressed, we have obtained transformants for all constructs injected so far (*n* > 35).

### sgRNA/Cas9 directed DNA cleavage

To obtain optimal cleavage efficiency, sgRNAs were designed to have a low GC content at the 5′end and high GC content in the seed region. sgRNA were further analysed for secondary structure and only those were chosen which do not disrupt the tracrRNA secondary structure. RNA secondary structure of sgRNAs was analyzed with RNAfold at http://rna.tbi.univie.ac.at (42). All sgRNAs were generated by in vitro transcription with T7 polymerase from synthetic oligonucleotides (0.2 μM) and trace-labeled with ^32^P alpha-ATP (800 Ci/mmol, 12.5 μM, Perkin Elmer) according to the manufacturer's instructions (Ambion). After DNAse I digestion, free nucleotides were removed with a G-50 Probequant Sephadex spin column (GE). Then, sgRNAs were heated for 2 min to 95°C and briefly left at room temperature to adopt folding, quantified by scintillation counting and analysed on 8% denaturing polyacrylamide gels.

To reconstitute synthetic oligonucleotide substrates, either a T7 promoter oligonucleotide (CCTGGCTAATACGACTCACTATAG) was annealed to an anti-sense Ultramer (IDT DNA) encoding the entire sgRNA in addition to the T7 promoter, or alternatively, a 60 nt T7 promoter oligonucleotide with a partial sgRNA was annealed to an anti-sense oligonucleotide encoding the tracrRNA (AAAAAAAGCACCGACTCGGTGCCACTTTTTCAAGTTGATAACGGACTAGCCTTATTTTAACTTGCTATTTCTAGCTCTAAAAC) for 15 min at 40°C (2 μM) and made double-stranded by extension with Klenow fragment of DNA polymerase I according to the manufacturer's instructions (NEB). Klenow was then heat-inactivated 10 min at 85°C and oligonucleotides were desalted with a G-50 Autoseq Sephadex spin column (GE) before using for in vitro transcription.

For sgRNA/Cas9 cleavage assays, DNA/sgRNA/Cas9 ratios of 1/10/10 were used in a 10 μl reaction using the buffer supplied (NEB) and DEPC-treated water. Typically Cas9 (100 nM final) was incubated with sgRNA (100 nM) for 10 min at 25°C before adding plasmid DNA (10 nM, corresponds to ∼25 ng/μl final concentration of a 3 kb plasmid). Plasmids were linearized after Cas9 digestion by first heat inactivating Cas9 for 2 min at 95°C, and then adding 10 μl of a restriction enzyme (5 U) in NEB buffer 3. Cleavage of plasmid DNA was analysed on ethidium bromide stained agarose gels.

## RESULTS

### Analysis of *Dscam* variable exon 4 inclusion frequency by a combination of RT-PCR, restriction enzyme digestion and denaturing acrylamide gel electrophoresis

Each of the variable exons in *Drosophila Dscam* clusters 4, 6 and 9 have about the same size and cannot be distinguished on agarose gels after RT-PCR amplification (Figure 1B). Sequences analysis of variable exons reveals, however, that they differ enough to find unique restriction sites to cut each exon into fragments of different length (Figure 1C). Accordingly, a combination of frequently cutting MboI, AluI, HinPI and TaqI restriction enzymes generates fragments of unique sizes for each individual exon in the *Dscam* exon 4 cluster. By including a ^32^P radioactive label in the forward primer, all 12 fragments can be visualized by separation in a single lane of sequencing-type denaturing acrylamide gels (Figure 1C and D), and are identified based on their predicted size, and match to the size from digests with individual enzymes (Figure 1D, compare lanes 2–5 with lane 6).

### Inclusion frequency of *Dscam* variable exons 4 varies only marginally during development or between individual flies

Applying this novel method to the analysis of inclusion levels in the *Dscam* exon 4 cluster during development, and in male and female adults revealed little variation with the exception of exon 4.9, which is included little, and exons 4.2 and 4.5, which are included more in adult flies (Figure 1E). Between individual males inclusion levels of exon 4 variables also differed little except for exon 4.4 (Figure 1F, Supplementary Figure S1). The lack of dynamics in the inclusion levels of exon 4 variables suggests a robust selection mechanism of individual variables likely directed by regulatory sequences in or around each variable.

### Generation of *GAL4/UAS* driven genomic reporter transgene for the analysis of alternative splicing

To decipher the mechanism responsible for mutually exclusive AS and selection of individual exons in the *Dscam* variable clusters, a reporter gene system is required, which recapitulates endogenous regulation and which can be efficiently manipulated despite its large size.

**Figure 2.**
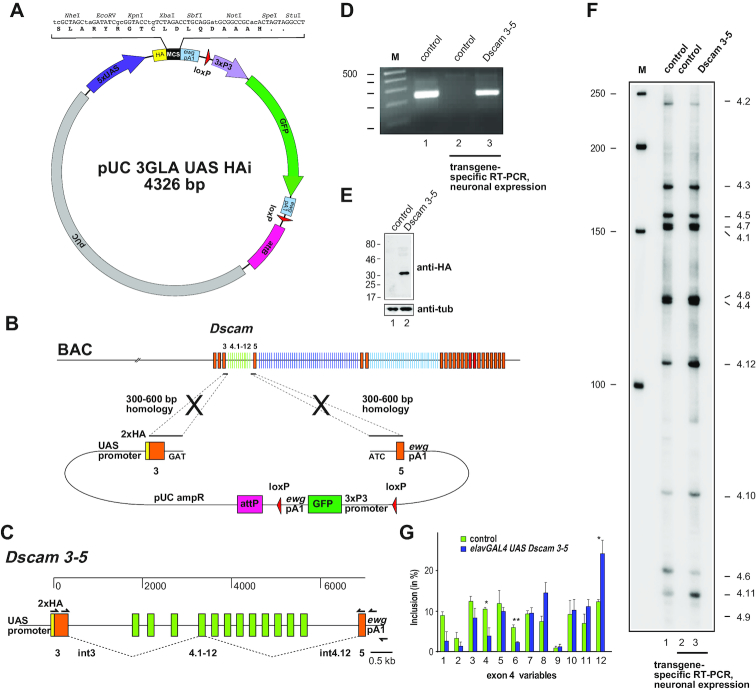
A reporter transgene for the analysis of *Dscam* mutually exclusive alternative splicing. (**A**) Schematic of the *pUC18* based high-copy number plasmid *pUC 3GLA UAS HAi* for *Drosophila* phiC31 transgenesis via the attB site (pink). The multiple cloning site (MCS, black) is shown on top indicating the reading frame for N-terminal tagging with a 3xhemagglutinin tag (HA, yellow). Heterologous expression is driven from the *5xUAS* promoter (purple) by the *GAL4* transcriptional activator and transcripts terminate at the polyA site from the ewg gene (light blue). The GFP marker (green) to identify transformed flies is driven by the artificial *3xP3* promoter (light purple) and flanked by *loxP* sites (red). (**B**) Schematic for the retrieval of the *Dscam* exon 3–5 region from a BAC containing the genomic locus using λ Red protein mediated gap-repair recombineering with a linearized plasmid containing homologous sequences of the beginning and the end of the construct. Homologous regions for recombination are indicated by crosses. (**C**) Graphical depiction of the *Dscam* exon 4 construct indicating primers for amplification of construct-specific transcripts using an RT primer in the *ewg* 3′UTR and nested PCR. (**D**) RT-PCR products for *Dscam* exon 4 shown on an agarose gel from the endogenous gene (lane 1) and from construct specific nested RT-PCR from control (lane 2) and transgenic flies with the *Dscam 3–5* construct neuronally expressed with *elavGAL4* (lane 3). (**E**) Western blot showing expression of the artificial Dscam 3–5 protein expressed with *elavGAL4* in larval brains and detected with anti-HA antibodies (lane2), compared to wild type controls (lane 1). (**F**) Denaturing acrylamide gel showing inclusion of individual exon 4 variable exons from the endogenous gene (lane 1), and from construct specific nested RT-PCR from control (lane 2) and transgenic third instar larval brains from neuronal expression of the *Dscam 3–5* construct with *elavGAL4* (lane 3). Note that the endogenous *Dscam* gene is more broadly expressed and therefore shows slightly different inclusion levels for some exons. (**G**) Inclusion levels of exon 4 variables in larval brains of controls (green) and from transgenic expression of *Dscam 3–5* from *UAS* in neurons using *elavGAL4* (blue) shown as means with standard error from three experiments. Statistically significant differences are indicated above bars (*P < 0.05).

Therefore, we constructed a minimal transformation vector for *Drosophila* transgenesis termed *pUC 3GLA HAi* based on the commonly used *UAS* expression vector *pUAST* now allowing inserts of up to 18 kb (Figure 2A). This ampicilin-resistant *pUC18* based minimal transformation vector contains a strong consensus ribosome entry site from the *Adh* gene, a hemagglutinin (HA) epitope-tag and a multiple cloning site (MCS) followed by a short 3′ UTR from the *erect wing* (*ewg*) gene (Figure 2A) (43). For transgenesis, an *attB* site for phiC31 transformation and a GFP marker expressed by the short artificial *3xP3* promoter were included. To avoid interference with transcription of the insert the GFP marker is placed after the *UAS* expression cassette. The GFP cassette is flanked by *loxP* sites to prevent interference with frequently used FRT sites in *Drosophila* and allows for later removal if required (44). The heterologous *UAS* promoter will allow for expression of the reporter gene in the required tissue by combining it with a tissue-specifically expressed *GAL4*.

Since most fly genes are smaller than 18 kb and usually have short well defined promoters, a genomic fragment can be cloned into the predecessor plasmid *pUC 3GLA* to generate a rescue reporter construct (Supplementary Figure S2). Analysis of AS in mutated derivatives of this rescue reporter construct requires placing it into the genetic background of an RNA null allele. This approach has the advantage to reveal phenotypes associated with defective AS regulation.

### A *Dscam* exon 3–5 reporter transgene recapitulates inclusion frequencies of variables in the exon 4 cluster

Next, we wanted to test if the genomic sequence from *Dscam* exon 3 to exon 5 harbors all regulatory elements and can recapitulate mutually exclusive AS when expressed from a heterologous *UAS* promoter. To recombine the *Dscam* exon 3–5 sequence into *pUC 3GLA UAS HAi*, we cloned homology regions from either side such that a unique EcoRV site was generated by fusing the two genomic fragments to allow for linearization of this plasmid (Figure 2B).

The linearized plasmid was then transfected into *E. coli*, which harbor a BAC with the *Dscam* gene and express the λ Red recombination proteins from a previously transfected plasmid, to retrieve the *Dscam* sequence between the homology arms (Figure 2B and C). When using 300–600 bp homology regions on either side, retrieval of DNA sequences by recombineering generally occurs with very high frequency for different genes (60–90%, n>8).

The *Dscam 3–5* construct was then injected into embryos expressing the phiC31 integrase in the germline and harboring an *attB* containing landing site for integration of the construct into the genome. For plasmids not exceeding a size of ∼20 kb, transformants are typically found in the progeny of one out of four to six fertile G0 flies (*n* > 35).

The transgene of the *Dscam 3–5* construct was then crossed to *elavGAL4* for neuron-specific expression. To analyse the transcripts specifically from this construct, we used a gene-specific primer in the *ewg 3*′*UTR* for reverse transcription (RT), and two rounds of nested PCR to only amplify *Dscam* from the construct, but not the endogenous locus (Figure 2D). Correct processing is further indicated by expression of a 29 kDa protein containing one variable of 6 kDa from the *Dscam 3–5* construct (Figure 2E).

Next, we analysed AS of the *Dscam 3–5* construct in neurons of third instar larval brains using restriction digest and separation on denaturing polyacrylamide gels (Figure 2F). The *Dscam 3–5* reporter transgene recapitulates mutually exclusive splicing and inclusion of all variable exons (Figure 2G). Compared with the alternative splicing pattern of endogenous *Dscam* gene in the whole brain, the *Dscam 3–5* reporter transgene largely recapitulates inclusion levels of the different variable exons, though significant differences were observed in variable exons 4.4, 4.6 and 4.12. These differences are likely due to neuron-specific expression of the construct compared to a broader expression of the endogenous gene, but are unlikely due to the vector composition as these exons are in the middle part of the construct. Hence, expressing the *Dscam* exon 3–5 region from a heterologous *UAS* promoter recapitulates mutually exclusive AS.

### Plasmid–based gap-repair recombineering

**Figure 3.**
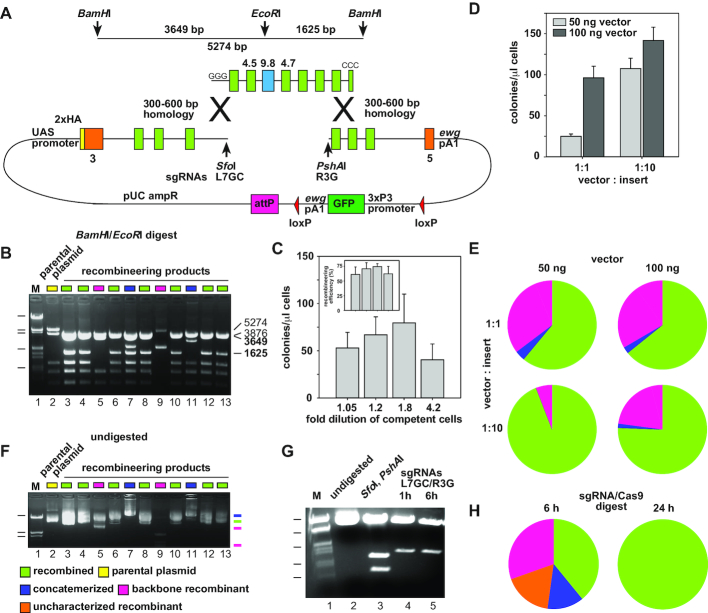
Plasmid based gap repair recombineering for modification of large plasmids. (**A**) Schematic for λ Red protein mediated gap-repair recombineering of the *pUC 3GLA HAi Dscam 3–5* plasmid linearized with SfoI and PshAI with a modified insert containing homologous sequences of the beginning and the end of the insert and vector. GGG at the beginning and CCC at the end of the insert indicate the genomic sequence of the SmaI sites used for excission from the *pOT2* vector (not shown). On top, fragment sizes for fingerprinting the parental and recombineered constructs with BamHI and EcoRI are indicated. Homologous regions for recombination are indicated by crosses. (**B**) Agarose gel of representative recombinant plasmids fingerprinted by BamHI/EcoRI restriction digests. Correct recombinants (green squares) are identified by 3649 and 1625 bp fragments originating from the 5274 bp fragment in the parental vector due to the additional EcoRI site introduced by exon 9.8. The parental plasmid is indicated by yellow, backbone recombinants by pink, uncharacterized recombinants by orange, and concatemerized plasmids by blue squares. Size markers are EcoRI/HinDIII digested λ DNA of 20, 5.2, 3.5, 1.9 and 0.8 kb. (**C**) Effect of competent cell concentration on transformation and recombineering efficiency. Transformation efficiency is shown as mean with standard error for the number of colonies obtained per microliter of competent cells (normalized to a transformation efficiency of 10^6^ transformants/μg of a 3 kb plasmid) from at least four experiments. Dilutions of the starting 25 μl of competent cells with 1.25 μl (1.05-fold dilution), 5 μl (1.2-fold), 20 μl (1.8-fold) and 80 μl (4.2-fold) are shown at the bottom. The insert shows the recombineering efficiency as percentage of positive clones using 50 ng vector with a vector to insert ratio of 1:10. (**D**) Effect of vector concentration and vector to insert ratio on transformation efficiency. Transformation efficiency is shown as mean with standard error for the number of colonies obtained per microliter of competent cells (normalized to a transformation efficiency of 10^6^ transformants/μg of a 3 kb plasmid) for 50 ng (light gray) or 100 ng of vector (dark gray) and a vector to insert ratio of 1:1 or 1:10 from at least eight experiments each and a total of 372 clones. (**E**) Effect of vector concentration and vector to insert ratio on recombineering accuracy and efficiency. Results are shown as pie charts from at least four independent experiments analyzing a total of at least 50 clones each for 50 ng (left) or 100 ng vector (right) and a vector to insert ratio of 1:1 (top) or 1:10 (bottom). (**F**) Agarose gel of undigested plasmids shown in (B). Colored lines on the right side indicate positions of supercoiled plasmids. Size markers are EcoRI/HinDIII digested λ DNA of 20, 5.2 and 3.5 kb. (**G**) Agarose gel showing Cas9 mediated cleavage of the Dscam 3–5 plasmid with sgRNAs L7GC and R3G. (**H**) Extended digestion with sgRNA/Cas9 is required for full plasmid cleavage. Results are shown as pie charts from two independent experiments with 6h and 24 h digestion time with sgRNAs L7GC and R3G using 50 ng vector and a vector to insert ration of 1:10 for transformation.

Given the high frequency for retrieving sequences from BAC clones by gap-repair we anticipated that we could use a similar approach to introduce mutations into large plasmids. The main requirement for this approach is the ability to introduce gaps, which can be done by using unique restriction sites present even in large plasmids, or by employing sgRNA/Cas9 directed endonucleolytic cleavage (45,46). Since Cas9 cleavage is directed by the sequence homology of the sgRNA to the target region that only requires two guanosines next to the cleavage site (protospacer adjacent motif, PAM), sgRNA/Cas9 cleavage can be directed close to the site of manipulation. Then, the gap in the plasmid can be closed by co-transfecting a piece of DNA with overlapping sequences on either side into *E. coli* containing recombineering proteins (Figure 3A).

In the first instance, we used restriction enzymes SfoI and PshAI to generate a gap for swapping exon 4.6 in *Dscam 3–5* with variable exon 9.8 from the exon 9 cluster. The complementary piece of DNA with 300–600 bp homology arms on either side was cloned into chloramphenicol-resistant *pOT2* vector (BDGP) by generating a SmaI site on either end to release the fragment with ends matching exactly the genomic sequence later. Exon 4.6 was then exchanged by exon 9.8 using overlapping PCR products and standard cloning techniques.

We then generated competent *E. coli* containing the recombination proteins and transfected them with an equimolar amount of vector and insert using 100 ng vector and 40 ng insert initially. A typical recombineering experiment resulted in about 60% correct clones, which were identified with BamHI and EcoRI fingerprinting restriction enzyme digests resulting in 3649 and 1625 bp fragments in the presence of exon 9.8 (Figure 3A and B). Once the gap is closed, any further recombination is rare and we generally did not observe reversion to the parental *pUC 3GLA HAi Dscam 3–5* plasmid (Figure 3B). To ensure high efficiency of plasmid-based gap-repair of large plasmids we used electro-competent cells of highest quality (39).

As detailed below a number of factors affect the outcome of the plasmid-based gap-repair recombineering. First, the quality of the competent cells critically determines the number of colonies that are obtained, but recombineering efficiency seemed less affected and yielded ∼50% correct clones for several batches of competent cells with efficiencies ranging from 1 × 10^5^–10^6^ transformant/μg 3 kb plasmid (*n* = 5). Indeed, for a given batch of competent cells, a 4-fold dilution resulted in minimal loss of transformation and recombineering efficiency (Figure 3C). We noticed, however, that a 30 min incubation of the fragments at 50°C prior to transformation increased the number of colonies about 2.5-fold by maintaining an efficiency of correct clones above 50% compared to incubation at 37°C or direct transformation (*n* = 3 each).

The other critical parameters for plasmid-based gap-repair recombineering are the concentrations of the vector used for transformation and the ratio of vector to insert. When comparing the recombineering efficiency of 50 ng vector with 100 ng vector at a vector to insert ratio of 1:1, we observed about a two-fold increase in colonies relative to the DNA concentration with about 60% correct clones each (Figure 3D and E). When using a 1:10 vector to insert ratio, the number of colonies increased about four fold for 50 ng vector, but only marginally for 100 ng vector (Figure 3D). In contrast, however, recombineering efficiency for 50 ng of vector and a 1:10 ratio of insert to vector resulted in over 90% correct clones (Figure 3E). Sequencing of both homology regions in 20 positive clones did not reveal any single point mutation indicating a very low error rate. In summary, a low amount of vector and a higher amount of insert is the preferred condition for plasmid-based gap-repair recombineering, which can occur with a high frequency (>90%) and high fidelity.

During plasmid-based gap-repair recombineering, aberrations can occur that need to be recognized by fingerprinting the plasmid with restriction digests yielding as many distinguishable fragments as possible (Figure 3B). By far the most prominent aberration we observed was recombination of the backbone vector, which was particularly frequent with a vector to insert ratio of 1:1 (Figure 3B and E). Despite the highly repetitive sequences in the *Dscam* exon 4 variable cluster, clones with aberrations in the insert were only observed at low frequency, indicating that plasmid-based gap-repair recombineering is also highly sequence-specific. One further class of aberrations are clones which have undergone concatemerization (Figure 3F). We noticed, that a further increase of vector to 500 ng considerably increased the occurrence of concatemerized plasmids, particularly when the insert to vector ratio was also increased (data not shown).

Gibson assembly provides an advance over traditional cloning as the sequence overlap between fragments is larger (∼20–24 nucleotides) and promises higher efficiency in manipulating plasmids (40). In addition, GA has the advance that fragments for cloning generally do not need to be gel-purified. We therefore compared cloning efficiency and fidelity of the Gibson assembly method with our recombineering method. Using Gibson assembly for the same plasmid and insert, we did get recombinant clones, however with a dramatically reduced cloning efficiency (<0.1%) compared to recombineering (Supplementary Figure S3). Further, equimolar ratios of fragments are instrumental for maximal efficiency. In addition, sequencing revealed a high error rate (up to 30%, *n* = 40 clones).

Next, we tested, whether sgRNA/Cas9 can cut with 100% efficiency to make it a suitable tool for efficient cloning. We chose to direct sgRNAs to two sequences in the proximity of the previously used SfoI and PshAI sites. These sgRNAs (L7GC and R3G) were in vitro transcribed from synthetic oligonucleotides harboring a T7 promoter and in combination with recombinant Cas9, cleave the *Dscam 3–5* plasmid at the expected sites (Figure 3G). Efficient gap-repair recombineering for such cleaved plasmids was successful, but required an extended digestion of 24 h as after 6h only about one third of the clones were recombinant, and after 1 h cleavage all clones tested were parental (Figure 3H, Supplementary Figure S4A-C). During extended digestions, we did not observe degradation of sgRNAs by spuriously present RNAses, likely because of protection by Cas9 (Supplementary Figure S4D).

We then explored, if a single cut either by L7GC or R3G sgRNA would suffice for successfully swapping exon 4.6 with exon 9.8, or if these would lead primarily to aberrant clones including concatemerization. Both L7GC or R3G sgRNA linearized the *Dscam3–5* plasmid and led to successful recombineering without favoring concatemerization (Supplementary Figure S4E–H).

### Exonic sequences are main determinants for the level of inclusion of *Dscam* exon 4 variables

**Figure 4.**
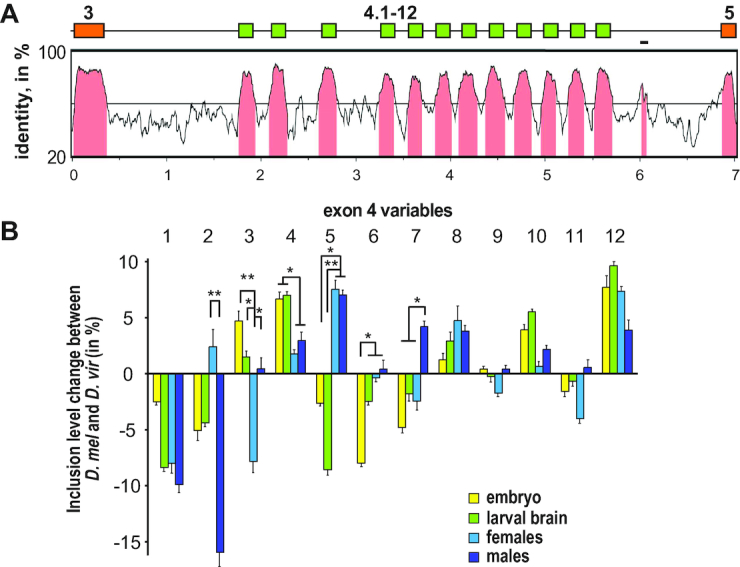
Phylogenomic analysis of the *Dscam* exon 4 cluster. (**A**) Vista plot alignment of *Dscam* exons 3 to 5 of the *D. melanogaster* sequence compared to the *D. virilis* sequence. Exons are shown as boxes on top and indicated in pink on the alignment. The line in intron 4.12 indicates the sequence assigned docking site by Yang *et al.* (47). (**B**) Developmental profile of differences in inclusion levels of exon 4 variables in embryos (yellow), third instar larval brains (green), adult females (dark blue) and males (light blue) of *D. melanogaster* compared to *D. virilis* shown as means with standard error from three experiments. Statistically significant differences are indicated above bars (*P < 0.05, **P < 0.01).

To determine regulatory mechanisms involved in selection of *Dscam* exon 4 variables, we aligned the sequence from *D. melanogaster* with the closely related species *D. virilis* to identify conserved sequence elements. This analysis showed that exonic sequences are very similar, while intronic sequences diverge considerably (Figure 4A). In intron 4.12 a conserved element is present, which had previously been assigned docking site for base-pairing with selector sequences in introns between the variable exons 4 (47). Re-analysis of introns in the exon 4 cluster for the presence of such selector sequences in *D. melanogaster* and *D. virilis*, however, only showed sequences in few introns, that would base-pair with the proposed docking site (Supplementary Figure S5).

**Figure 5.**
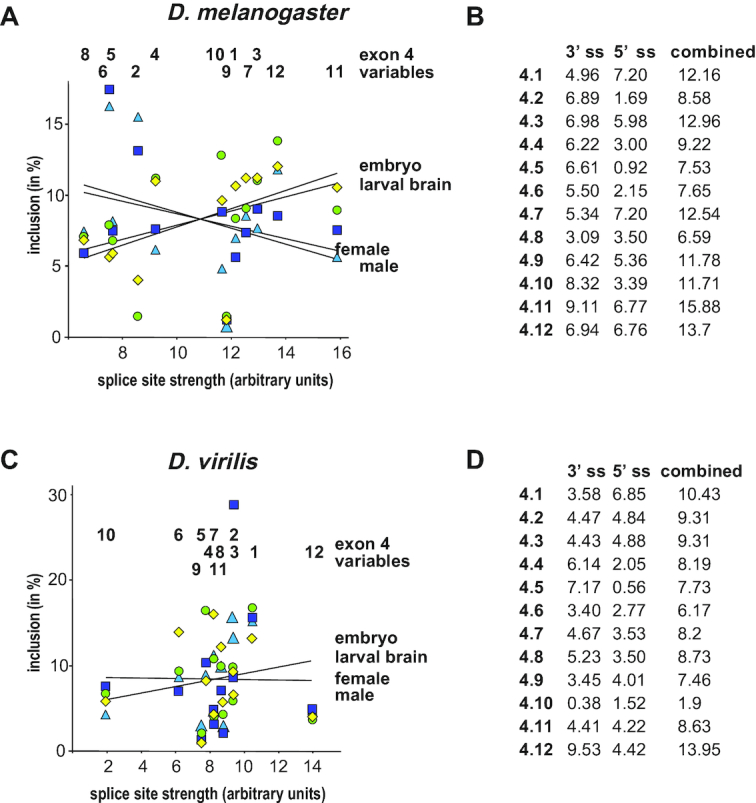
Splice site strength of *Dscam* exon 4 variables does not correlate with inclusion levels. (**A**) Splice site strength of *D. melanogaster* exon 4 variables are plotted against the mean of inclusion levels from three experiments during development (embryos in yellow and third instar brains in green) or in adult females (dark blue) and males (light blue). Trends for different samples are indicated as lines. (**B**) Scores for 5′, 3′, and combined splice sites for *Dscam* exon 4 variables from *D. melanogaster*. (**C**) Splice site strength of *D. virilis* exon 4 variables are plotted against the mean of inclusion levels with standard error from three experiments during development (embryos in yellow and third instar brains in green) or in adult females (dark blue) and males (light blue). Trends for different samples are indicated as lines. (**D**) Scores for 5′, 3′, and combined splice sites for *Dscam* exon 4 variables from *D. virilis*.

Inclusion levels of exon 4 variants are largely similar during development and in adults in both species with mostly small differences of which some are statistically significant (Figure 4B and Supplementary Figure S6) indicating that either the proximity of ss sequences to the consensus (splice site strength), or regulatory elements in exons determine inclusion levels. Therefore we analyzed how well ss sequences of variable exons match the consensus since inclusion levels of exons generally correlate with ss strength (for refs see (12). In neither species, we detected a correlation of inclusion levels with ss strength (Figure 5A–D). These results argue that regulatory elements within exons harbor determinants for inclusion.

**Figure 6.**
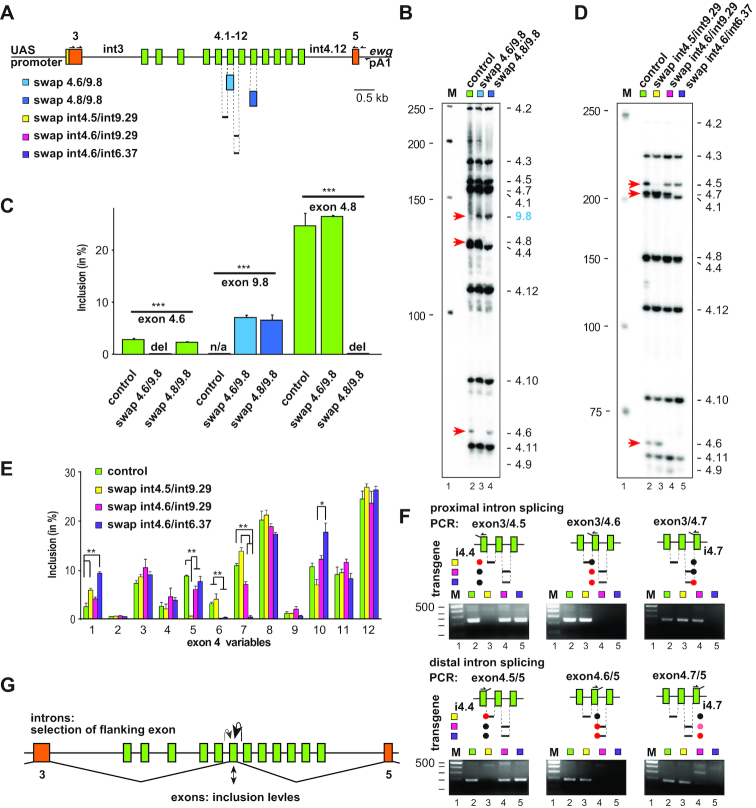
Exon and intron sequences determine inclusion levels in the *Dscam* exon 4 cluster (**A**) Schematic of the *pUC 3GLA HAi Dscam 3–5* depicting transgenes ‘swap 4.6/9.8’ (light blue) and ‘swap 4.8/9.8’ (dark blue) where exon 4.6 or 4.8 was exchanged with exon 9.8, respectively. ‘Swap int4.5/int9.29’ (black line), ‘swap int4.6/int9.29’ (black line) and ‘swap int4.6/int6.37’ (grey line) indicate transgenes with accordingly exchanged introns. (**B**) Denaturing acrylamide gel showing inclusion of individual exon 4 variable exons from neuronal expression with *elavGAL4* in third instar larval brains from construct specific nested RT-PCR and identification by restriction digests (MboI, AluI, HinPI and TaqI) from control (*Dscam 3–5*, green, lane 2), the exchange of exon 4.6 with exon 9.8 (swap 4.6/9.8, light blue, lane 3) and exon 4.8 with exon 9.8 (swap 4.8/9.8, dark blue, lane 4) transgenes. Red arrowheads point towards absent exons and the blue arrowhead points towards exon 9.8 swapped for exon 4.6 or 4.8, respectively. (**C**) Quantification of inclusion levels for exons 4.6, 4.8 and 9.8 in the control (*Dscam 3–5*), swap 4.6/9.8 and swap 4.8/9.8 transgenes shown as means with standard error from three experiments. Statistically significant differences are indicated above bars (**P < 0.001). (**D**) Denaturing acrylamide gel showing inclusion of individual exon 4 variable exons from neuronal expression with *elavGAL4* in third instar larval brains from construct specific nested RT-PCR and identification by restriction digests (MboI, AluI, HinPI and TaqI) from control (*Dscam 3–5*, green, lane 2), the exchange of intron 4.5 with intron 9.29 (swap int4.5/int9.29, yellow, lane 3), intron 4.6 with intron 9.29 (swap int4.6/int9.29, magenta, lane 4) and intron 4.6 with intron 6.37 (swap int4.6/int6.37, purple, lane 5) transgenes. Red arrowheads point towards absent exons. (**E**) Quantification of inclusion levels for exons 4.1 to 4.12 in control (*Dscam 3–5*, green), swap int4.5/int9.29 (yellow), swap int4.6/int9.29 (magenta) and swap int4.6/int6.37 (magenta) transgenes shown as means with standard error from three experiments. Statistically significant differences are indicated above bars (*P < 0.05, **P < 0.01). (**F**) Diagnostic PCR from cDNA of each transgene for splicing of the proximal (top) and distal (bottom) intron of indicated variable exons. Primers are indicated above exons and swapped introns below according to the color code: swap int4.5/int9.29 (yellow), swap int4.6/int9.29 (magenta) and swap int4.6/int6.37 (magenta). Black dots indicate productive splicing, pink dots weak splicing and red dots mark absent splicing. PCR products were separated on 3% agarose gels. One hundred base pair size markers are shown on the left. (**G**) Model for the regulation of mutually exclusive alternative splicing in the *Dscam* exon 4 cluster. Intron sequences between exon 4 variables are key to selection of preceding variable exons (black arrow), and to a lesser extent for selection of the following exon (grey arrow) as indicated on top of the gene model. Sequences within variable exons impact on their inclusion levels (double arrow, bottom).

To test this hypothesis, we generated transgenes by phiC31 integrase-mediated integration for two constructs where exons 4.6 and 4.8 were replaced by 9.8, respectively (Figure 6A). Exons 4.6 and 4.8 were chosen, because they show the lowest and highest inclusion levels from the *Dscam 3–5* reporter in neurons, respectively, and are localized in the regularly arrayed part of the cluster (Figure 2 F and G). After crossing these lines to *elavGAL4*, alternative splicing was analyzed in neurons of third instar larval brains by transgene-specific RT-PCR, digestion with restriction enzymes and resolving the fragments on denaturing polyacrylamide gels (Figure 6B). The general pattern for inclusion of *Dscam* exon 4 variable exons was very similar, except that exons 4.6 and 4.8 were absent and replaced by exon 9.8 in the swap 4.6/9.8 and the swap 4.8/9.8 lines, respectively. Exon 9.8 is included equally in both lines while exon 4.6 has low and exon 4.8 high inclusion levels (Figure 6C). This result indicates that exon sequences are main determinants for inclusion frequency of variable exons in the *Dscam* exon 4 cluster.

### Intronic sequences are required for selection of *Dscam* exon 4 variables

It has been suggested that RNA secondary structure from base-pairing between the intron of the selected preceding exon (selector sequence) and a sequence in the last exon of the cluster (docking site) is important for mutually exclusive alternative splicing of *Dscam* exon 4 and 9 clusters by bringing ss into proximity (47). Our analysis of *D. melanogaster* and *D. virilis Dscam* exon 4 clusters, however, revealed little evolutionary conservation of selector sequences (Figure 4A and Supplementary Figure S5).

To test the role of introns in *Dscam* exon 4 cluster mutually exclusive alternative splicing we exchanged introns 4.5 and 4.6 with intron 9.29, and intron 4.6 with intron 6.37, which are similar in length, sequence composition and capacity to base-pair with the putative docking site (Supplementary Figure S5E). Also, swapped introns did not generate secondary structure in the new context that is qualitatively different from the normal context. After generating transgenes and crossing these lines to *elavGAL4*, alternative splicing was analyzed in neurons of third instar larval brains by transgene-specific RT-PCR, digestion with restriction enzymes and resolving the fragments on denaturing polyacrylamide gels (Figure 6D).

For all three transgenic lines with swapped introns, the exon preceding the swapped intron was not included, but for the intron 4.6/6.37 also the following exon was not included, and inclusion in the 4.6/9.29 was significantly reduced (Figure 6D and E). The general pattern for inclusion of *Dscam* exon 4 variable exons, however, was very similar for all three lines (Figure 6D and E).

To assess whether the swapped intron would affect splicing to constant exons on both distal and proximal sides, we performed diagnostic PCR with exon-specific primers for cDNAs of each transgene (Figure 6F). This analysis reveals that splicing on both sides of the variable exon preceding the swapped intron is absent (Figure 6F). In addition, the intron 4.6/6.37 swap also inhibited splicing of exon 3 to variable exon 4.7. Likewise, the intron 4.6/6.37 swap, and to a lesser extent the intron 4.6/9.29 swap inhibited splicing of variable exon 4.7 to exon 5 (Figure 6F).

Taken together, our data show that exon sequences are important to determine inclusion levels, while intron sequences are required for selection of the preceding variable exon, and to a lesser extent the following exon (Figure 6G).

## DISCUSSION

The large size of most introns in human genes makes it difficult to reveal the mechanisms involved in ss recognition and tissue-specific AS regulation at genomic scales (12). Here, we adopt a *Drosophila* model to elicit principles of tissue-specific AS regulation by developing a plasmid-based reporter platform for efficient manipulation of large plasmids. Using recombineering in *E. coli*, we demonstrate that plasmid-based gap-repair is a highly efficient and reliable method to introduce mutations into large plasmids circumventing the time-consuming and tedious introduction of selection cassettes and counter selection for desired mutations during manipulation of BACs or other large vectors.

We have tweaked our system to include the maximum DNA fragment size that can be included in high copy number plasmids, which have high integration rates during transgenesis. In addition, our approach is expandable to larger *P[acman]* vectors or other libraries available in *Drosophila* and other organisms (48). These larger vectors are maintained at low copy number in *E. coli* and production of high amounts of DNA for manipulation or transgenesis can be induced by a plasmid copy control mechanism, but the larger size results in lower integration rates during transgenesis (49).

The high efficiency of DNA manipulation by gap-repair recombineering requires introducing two double-strand cuts into the DNA. Generally, restriction enzymes can be identified that cut only once within a 20 kb plasmid, but such sequence limitations can now be overcome by the use of the sgRNA/Cas9 endonuclease. In addition, using the sgRNA/Cas9 endonuclease together with gap-repair recombineering will now also allow for efficient modification of larger vectors such *P[acman]*. DNA scission by the sgRNA/Cas9 complex is highly specific and requires complete base-pairing between the sgRNA and the target DNA generally not tolerating single miss-matches (50,51). Although this feature makes the sgRNA/Cas9 complex an ideal tool for DNA editing, limited predictability of efficient cleavage of targets might require optimization in selecting sgRNA target sequences.

As a model to demonstrate the reliability of plasmid-based gap-repair, we chose the highly repetitive *Dscam* gene as we anticipated that the complex nature of the *Dscam* variable exon 4 cluster would be a good sensor to reveal possible weaknesses. Although plasmid-based gap-repair is highly reliable and efficient, two points need attention.

First, we observed that higher concentrations of plasmids and inserts favored concatemerization. Such aberrations, however, can easily be recognized by analysing the size of undigested plasmids. Secondly, it is possible that positive clones contain contaminating plasmids due to the relatively high amounts of large linearized plasmids required for transfection. This issue, however, can be resolved during phiC31 integrase-mediated transgenesis. Here, only one molecule of the construct is integrated into the landing site during *attB/attP* recombination. Since transgenic lines are established from a single insertion event, it is principally possible to select the desired construct at the level of the transgenic lines obtained. From our experience, however, we haven’t come across a faulty transgene from over 35 constructs with this strategy. For phiC31 mediated integration into cell lines, however, it is advisable to reselect the plasmid in a second round of transforming bacteria to ensure a single plasmid species.

For tissue-specific expression of the *Dscam 3–5* reporter we have made use of the binary *GAL4/UAS* system widely used in Drosophila (31,33). This heterologous expression recapitulated the endogenous splicing pattern of *Dscam* exon 4. The minor differences observed can either be attributed to differences in tissue-specific splicing regulation, or could be mediated by increased expression levels from the *UAS* construct. Potential routes to better control expression levels could include inducible *GAL4*, the combination with a temperature sensitive *GAL80* inhibitor of *GAL4* or the use of endogenous promoters, but also choice of different genomic locations of landing sites can affect expression due to position effects. Since the concentration of RBPs is critical for the recognition of targets and the regulation of AS (13), this aspect certainly needs attention in the design of reporter systems. Although, we previously did not observe any effect of the promoter in regulating AS splicing of the *ewg* gene in *Drosophila* (14), such effects have been demonstrated in AS regulation of the *fibronectin* gene in cell culture (52).

The extraordinary molecular diversity generated by mutually exclusive AS in the *Dscam* gene is functionally relevant for wiring of the nervous system and in adaptive immunity (9). In the nervous system, neighboring neurons require *Dscam* diversity to bifurcate from axonal tracts and to generate overlapping dendritic fields. Although choice of a particular exon combination occurs stochastically during development, it can change during neuronal development and in the immune system (9,53). We observed relatively little variation for inclusion of *Dscam* exon 4 variables during development and in individual flies, which is comparable to previous studies (54–57). In addition, sequence conservation in introns of the variable cluster was low indicating absence of regulatory sequences relying on strict sequence conservation. These results argue that a specific exon is selected depending on regulatory sequences residing within a particular variable exon. Indeed, replacing variable exons in the *Dscam* exon 4 cluster with an unrelated exon sequence resulted in constant inclusion levels, which were different from the inclusion levels of the replaced exon 4 variables and which were independent of the position in the cluster (Figure 6G).

A model for *Dscam* mutually exclusive alternative splicing has been proposed whereby RNA secondary structure formed by base-pairing of selector sequences before (exon 6) or after the selected exon (exon 4 and 9) with docking sites in the beginning (exon 6) or the end (exon 4 and 9) of the cluster are key to bring ss into proximity for activating the ss of the selected exon for its inclusion in the mature transcript (47,58). Although considerable evolutionary sequence conservation has been found in the exon 6 cluster, such sequences in the exon 4 and 9 cluster are much less conserved and are also using docking sites at the end of the cluster raising the question whether exon 4 and 9 would use the same mechanism for mutually exclusive alternative splicing.

To test whether this model applies to the exon 4 cluster and whether selector sequences are present in introns, we swapped introns from the exon 4 cluster with introns of the exon 6 an 9 cluster. Since selector sequences in these intron are different, they should specifically exclude the preceding exon. Although swapping of introns resulted in the complete lack of inclusion of the preceding exon, which is consistent with the postulated model, we also found that splicing of the distal exons can be affected. This outcome is not predicted for the selector/docking site model. Also, swapping selector and docking sites in a mini-gene resulted in mixed results (47). In addition, we did not find extensive sequence conservation in introns of the exon 4 cluster, nor base-pairing between these introns and the conserved sequence in exon 4.12, that hasbeen termed docking site (47). Further, putative selector sequences for the exon 4 cluster were also detected in introns 6.37 and 9.29 (Supplementary Figure 5E). Likewise, if long-range base-pairing was key to exon 4 mutually exclusive alternative splicing by bringing ss together, we would expect that the docking site is close to the distal end of intron 4.12 and not just next to exon 4.12.

Therefore, the splicing defects we observed by swapping introns from the exon 4 cluster with introns from the exon 6 and 9 clusters are likely mediated by RNA binding proteins that act cluster specifically as has been described for the exon 6 cluster (59).

In conclusion, our data suggest a model whereby intron sequences between exon 4 variables are key to selection of preceding variable exons, and to a lesser extent for selection of the following exon. In addition, sequences within a variable exon impact on their inclusion levels (Figure 6G). Our data further argue against a model implementing selector-docking site base-pairing for variable exon selection as we did not find strong sequence conservation mediating such base-pairing. Also, a docking site in the last exon would likely require transcription of the entire variable cluster before a variable exon can be selected. In fact, our data from swapping intron 4.6 with 9.29, show that the proximal splicing from exon 3 to exon 4.6 occurred, while distal splicing was compromised suggesting that splicing of the proximal exon occurs before splicing of the distal exon, also requiring a stem-loop structure in intron 3 (9,58,60).

Splicing regulation is a highly complex, yet essential process for the expression of genes and current estimates indicate that half of human disease-causing mutations are associated with splicing defects (4,7). Here we have developed a highly efficient platform for the study of splicing mechanisms, which will in combination with transgenic model organisms aid the study of tissue-specific AS regulation, including the study of defective AS in the brain, to develop novel therapeutics to treat disease associated with AS defects (61).

## DATA AVAILABILITY

Plasmid sequences haven been deposited in Genbank: KM253740 (pUC 3GLA UAS HAi), KM977568 (pUC 3GLA) and KM977569 (pOT2 3GLA UAS HAi) and plasmids are available from Addgene.

## Supplementary Material

Supplementary Data
