# Supplementary material for: Plasmid-based gap-repair recombineered transgenes reveal a central role for introns in mutually exclusive alternative splicing in Down Syndrome Cell Adhesion Molecule exon 4
Source: Nucleic Acids Res. 2018 Dec 12;47(3):1389–403. doi: 10.1093/nar/gky1254 (PMC6379703; doi:10.1093/nar/gky1254)

Plasmid-based gap-repair recombineered transgenes reveal a central role for introns in mutually exclusive alternative splicing in *Dscam* exon 4

IRMGARD U. HAUSSMANN, PINAR USTAOGLU, ULRIKE BRAUER, YASH HEMANI, THOMAS DIX AND MATTHIAS SOLLER

Legends for Supplementary Figures

Supplementary Figure S1. Inclusion levels of individual exon 4 variables of ten individual males. Statistically significant differences are indicated above bars (*, $p<0.05$).

Supplementary Figure S2. Plasmid maps of pUC 3GLA (A) and pOT2 3GLA (B). pUC or OPT based fly transformation vectors containing an attB site and a loxP site flanked GFP marker expressed under the 3xP3 promoter for possible later removal.

Supplementary Figure S3. Comparison of plasmid based gap-repair recombineering with Gibson Assembly.

(A, B) Agarose gel of representative recombinant plasmids fingerprinted by BamHI/EcoRI restriction digests (top) or undigested (bottom) after Gibson Assembly using a vector to insert ratios of 1:1 (A) or 1:10 (B). Correct recombinants (green squares) are identified by 3649 bp and 1625 bp fragments originating from the 5274 bp fragment in the parental vector due to the additional EcoRI site introduced by exon 9.8. Backbone recombinants are indicated by pink squares. Size markers are EcoRI/HinDIII digested λ DNA of 20 kb, 3.6 kb, 1.9 kb and 0.8 kb.

(C) Summary of Gibson Assembly accuracy with vector to insert ratios of 1:1 (left) or 1:10 (right). Results are shown as pie charts from three experiments analyzing a total of at least 50 clones each using 100 ng vector. Correct recombinants are indicated in green, backbone recombinants in pink and plasmids with sequence errors in blue squares.

(D) Comparison of recombineering and Gibson Assembly efficiencies from three independent experiments.

Supplementary Figure S4. Plasmid based gap-repair recombineering after sgRNA/Cas9 mediated cleavage.

(A-C) Agarose gel of representative recombinant plasmids fingerprinted by BamHI/EcoRI restriction digests (top) or undigested (bottom) after 1h (A), 6h (B) or 24h (C) cleavage with sgRNAs L7GCand R3G and Cas9 endonuclease prior to recombineering. Correct recombinants (green squares) are identified by 3649 bp and 1625 bp fragments originating from the 5274 bp fragment in the parental vector due to the additional EcoRI site introduced by exon 9.8. The parental plasmid is indicated by yellow, backbone recombinants by pink, uncharacterized recombinants by orange, and concatamerized plasmids by blue squares. Size markers are EcoRI/HinDIII digested λ DNA of 20 kb, 3.6 kb, 1.9 kb and 0.8 kb.

(D) Denaturing polyacrylamide gel showing the integrity of sgRNAs before and after 24 h incubation.

(E) Agarose gel showing Cas9 mediated cleavage of the Dscam 3-5 plasmid with either sgRNA L7GC or R3G. Plasmids were cut with NotI after Cas9 mediated cleavage.

(F-H) Agarose gel of representative recombinant plasmids fingerprinted by BamHI/EcoRI restriction digests (top) or undigested (bottom) after 24h cleavage with either sgRNA L7GC (F)

or R3G (G) in combination with Cas9 endonuclease prior to recombineering, and summary shown as pie chart of outcome events (H). Correct recombinants (green squares) are identified by 3649 bp and 1625 bp fragments originating from the 5274 bp fragment in the parental vector due to the additional EcoRI site introduced by exon 9.8. The parental plasmid is indicated by yellow, backbone recombinants by pink, uncharacterized recombinants by orange, and concatamerized plasmids by blue squares. Size markers are EcoRI/HinDIII digested λ DNA of 20 kb, 3.6 kb, 1.9 kb and 0.8 kb.

Supplementary Figure S5. Alignment of introns between variable exons from the exon 4 cluster of *D. melanogaster* (A) and *D. virilis* (D), and analysis for sequences complementary to the conserved sequence from intron 4.12 termed docking site (B, Yang et al, 2011). Sequences with base-pairing capacity to the intron 4.12 sequence termed docking site are shown for *D. melanogaster* (C) and *D. virilis* (D), as well as introns 6.37 and 9.29 (E). Note that sequences with base-pairing capacity to the intron 4.12 sequence termed docking site are absent from most introns.

Supplementary Figure S6. Developmental profile of inclusion levels of exon 4 variables in embryos (yellow), third instar larval brains (green), adult females (dark blue) and males (light blue) of *D. virilis* shown as means with standard error from three experiments. Exons 4 and 12 can not be separated and are shown as sum. Statistically significant differences are indicated above bars (*, $p<0.05$, **, $p<0.01$, ***, $p<0.001$)

Supplementary Figure S1

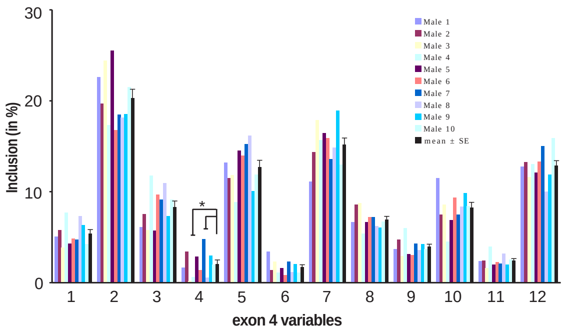

Supplementary Figure S2

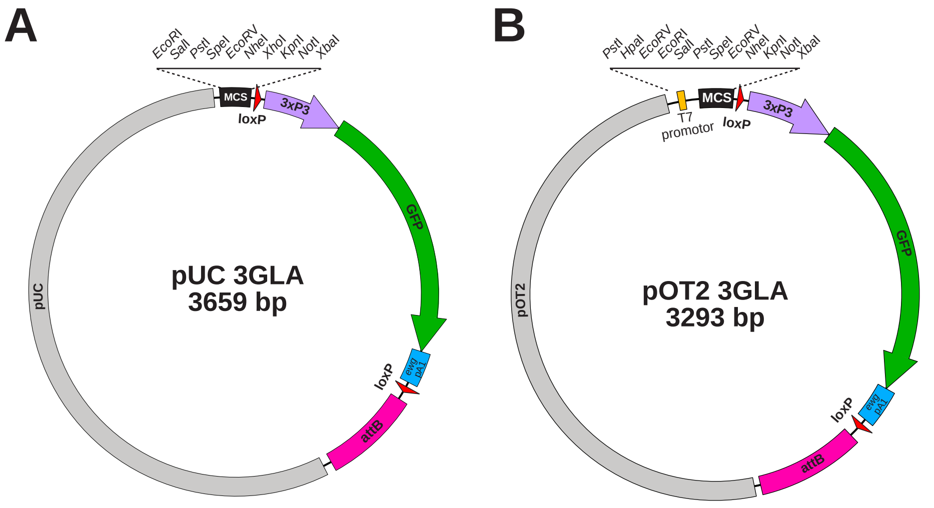

Supplementary Figure S3

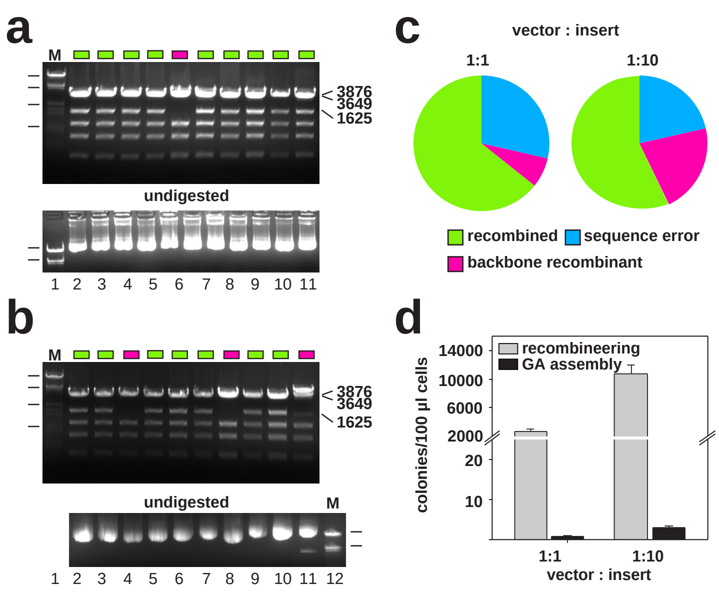

Supplementary Figure S4

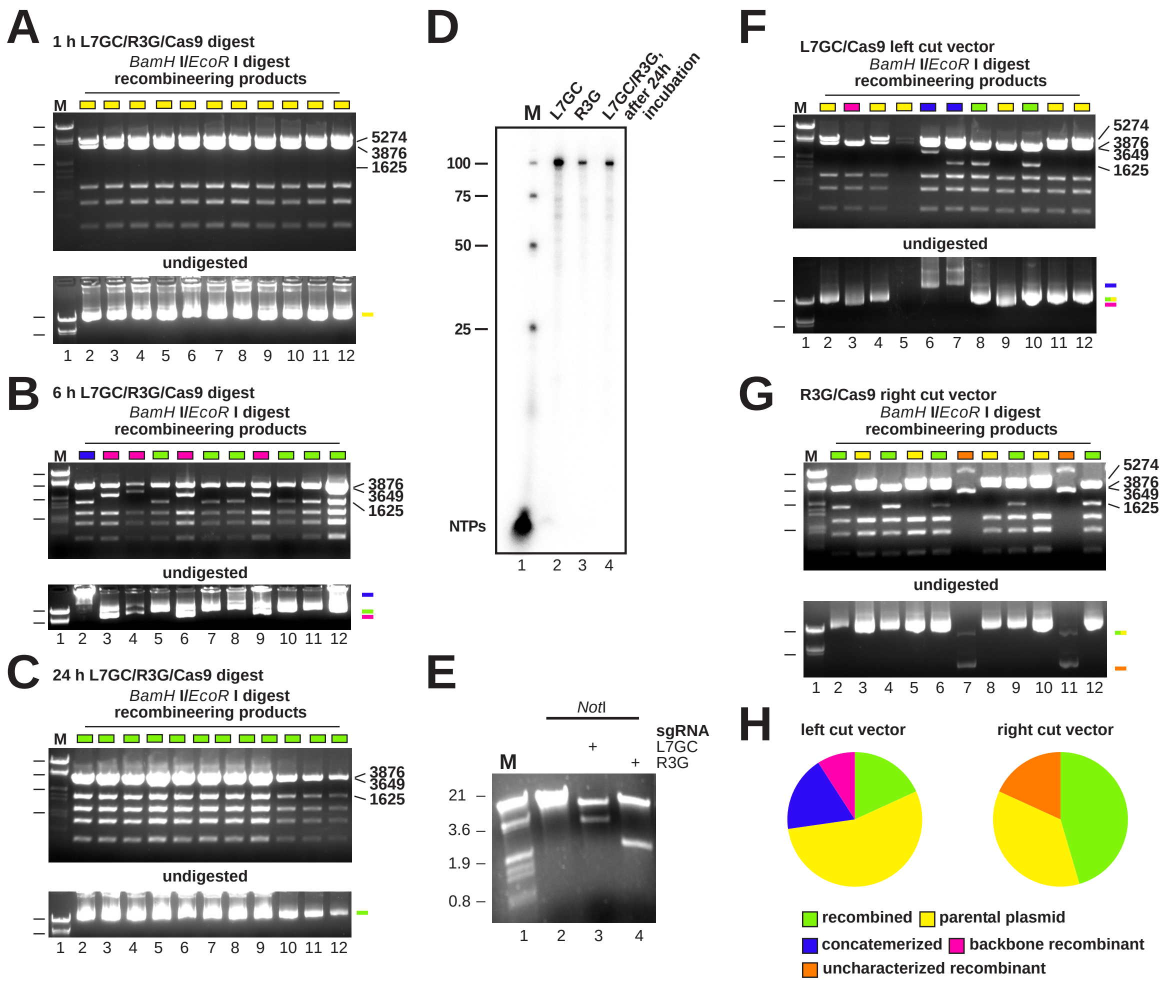

Supplementary Figure S5

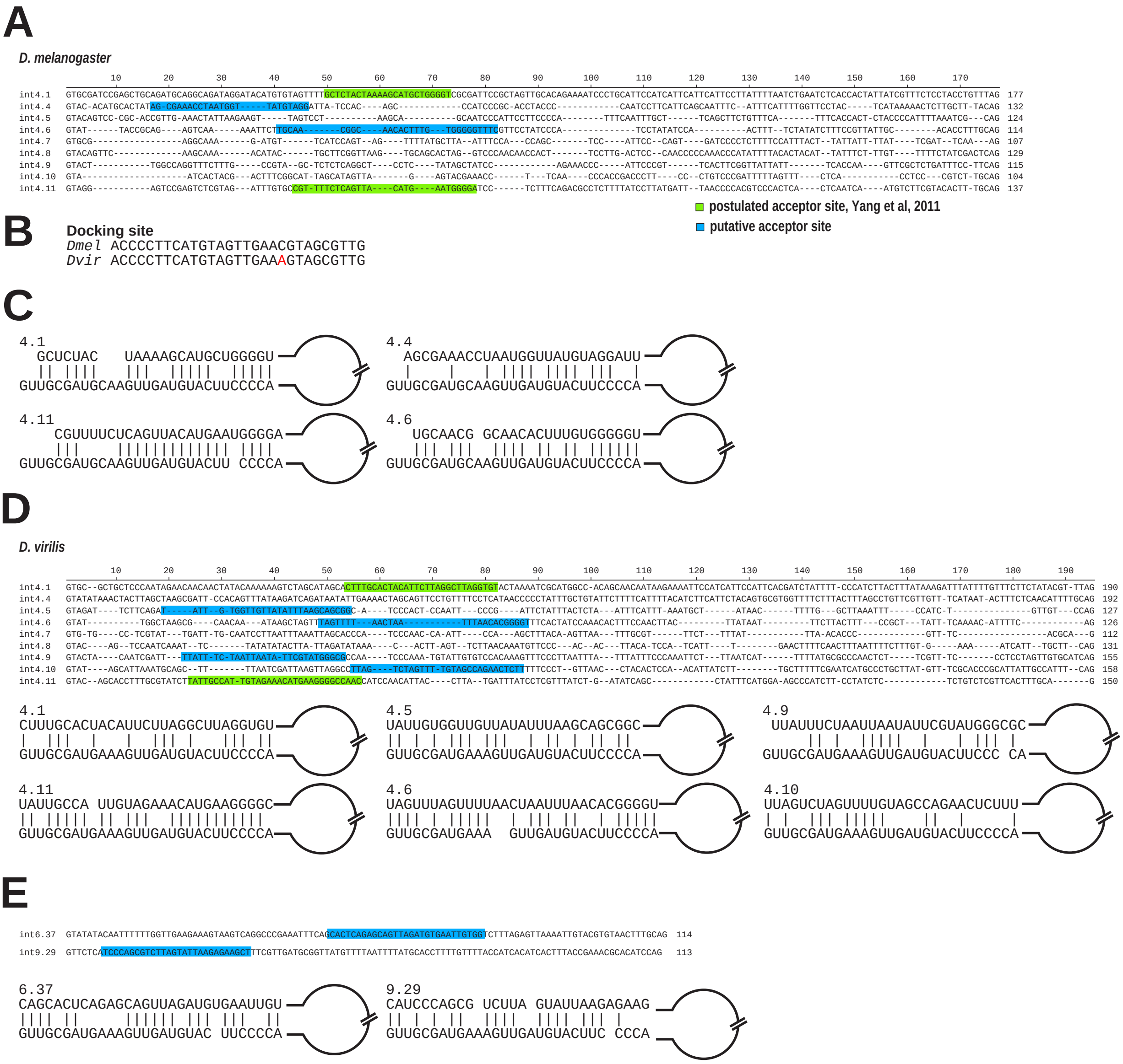

Supplementary Figure S6

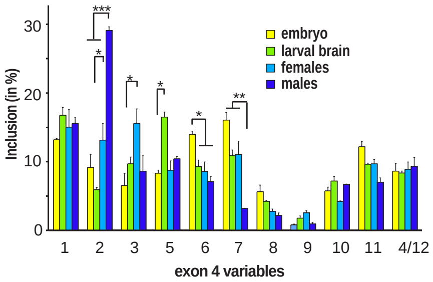

Supplement: Supplementary Data [file gky1254_supplemental_files.pdf]
